# Supplementary material for: Puppies Raised during the COVID-19 Lockdown Showed Fearful and Aggressive Behaviors in Adulthood: An Italian Survey
Source: Vet Sci. 2023 Mar 5;10(3):198. doi: 10.3390/vetsci10030198 (PMC10059587; doi:10.3390/vetsci10030198)
Supplement: Supplementary file 1 [file vetsci-10-00198-s001.zip › vetsci-2164760-supplementary.pdf]

Hello, thank you for your attention!

We are here to offer you a questionnaire: this is your contribution to our research activity, at a time of prolonged fragility and uncertainty caused by the pandemic.

Your help will be instrumental in assessing the impact of lockdown in the period March-May 2020 on the growth and behavioral development of your dog, following isolation caused by the outbreak by COVID-19 and how much this has been able to influence in the relational field.

Fill out the questionnaire if your dog was between 1 and 5 months during the period March-May 2020 or if your dog was born between June 2020 and February 2021.

This is a multidisciplinary study resulting from the collaboration between the Department of Veterinary Medicine and Animal Production of the University of Naples Federico II and the Department of Psychology of Processes of Development and Socialization of the University of Rome La Sapienza.

Filling out the questionnaire will take about 10 minutes and we want to make sure that it is completely anonymous: in no way will it be possible to trace it back to you.

The data will be used only in aggregate form and in full compliance with current privacy legislation, as required by Legislative Decree 101/2018.

Good participation!

Information on the processing of personal data the following questionnaire follows the privacy regulations established by Legislative Decree 10 August 2018, n. 101 adapting the Code on the protection of personal data (Legislative Decree 30 June 2003, n. 196) the provisions of Regulation (EU) 2016/679. Information pursuant to art. 13 of EU Regulation 679/2016 on the processing of personal data. The data treated with this questionnaire will be used exclusively for this purpose and in any case within the institutional purposes of the University of Naples Federico II. The data subject is entitled to the rights referred to in Articles 15-22 of the EU Regulation. The data subject. The data controller is the University in the persons of the Rector and the Director General, in relation to the specific competences. Exclusively in order to report any violations in the processing of your personal data, you can contact the Data Controller at the following address: ateneo@pec.unina.it; or the Data Protection Officer: rpd@unina.it, via pec: rpd@pec.unina.it. For information and/or clarification on this procedure, please write to: dip.medicina-veterinaria-prodan@pec.unina.it.

- I accept
- I do no accept

- Was the dog 1 to 5 months old during the March-May 2020 lockdown?

- Yes - No

- **Was the dog born between June and February 2021?**
    - Yes - No

OWNER INFORMATION

- **Age of owner in years**
  - 20-30 - 30-40 - 40-50 - 50-60 - over 70

- **Sex at birth**
  -Female - Male

- **Job**
  -Student - Employee - Work in the dog/ veterinary field - Freelance
  - Self employed - Retired - Other (Specify)

- **Level of education**
  -Primary School - High School - College

- **Composition of the family unit**
  -One component -Two components - Three components
  -Four components - Five components - Six or more components

- **How many children make up the household**
  -One children - Two childrens - Three childrens
  -Four childrens Five childrens Six or more childrens

HOME INFORMATION

- **Size of dwelling**

  -up to 65 square meters - from 66 to 110 square meters

  -from 110 to 150 square meters - Over 150 sqm
- **Presence of balconies and/or terraces?**

  -Yes - No
- **Presence of outdoor spaces (e.g. green areas, courtyards, etc.)?**

  - For common use - For private use

  - Both for common and Private use - No green zone
- **Can the dog enjoy the outdoor spaces?**

  -Yes - No

- **How many dogs live in the house?**

  - only the one adopted - that adopted more another dog

  - that adopted more two dogs - that adopted more three dogs
- **Indicates the age of the members of the dog's group**

  - 0-2 3-5 6-10 over 10 years
- **Are there other animals in the house?**

  -Yes (quantify which) - No
- **Sex of the dog**

  - Male - Female - Neutered - Intact
- **Dog weight**

  -Up to10kg - From 11to 20kg - Over 21kg
- **In what way did you adopt your dog?**

  -Adopted from the shelter

  - Adopted by another person

  - Founded as strays

  - Purchased by private

  - Purchased on the farm

  - Purchased in store

  - Other (Specify)
- **How old was the dog at the time of adoption?**

  -born at home - 1 month of life - 2 months of life

  - 3 months of life - 4 months of life - 5 months of life
- **Were there any problems managing the dog after adoption?**

  -Yes (when) - No
- **Did the dog attended a dog trainer center?**

  -Yes (indicate who followed him and when) -No
- **Does the dog suffer from organ pathology?**

  -Yes (which) - No

Here are a number of personality traits and behavioral descriptions that may or may not apply to your dog. Write a number next to each statement to indicate the extent to which you agree or disagree with that statement. You should evaluate your dog based on its behavior in general.

1= disagreeing strongly
2= disagreeing moderately
3= disagree slightly
4= neither in agree nor disagree
5= agree slightly
6= agree moderately
7= agree strongly

- Dog is relaxed when greeting people
- Dog behaves aggressively towards dogs
- Dog is anxious
- Dog is lethargic
- When off leash, dog comes immediately when called.
- Dog is shy
- Dog behaves aggressively towards unfamiliar
- people.
- Dog likes to chase birds or other small animals
- Dog gets bored quickly in play
- Dog is quick to sneak out through open doors or gates
- Dog is confident
- Dog is dominant towards other dogs
- Dog avoids other dogs
- Dog performs tasks until completed (for example, getting treats from a Kong)
- Dog is boisterous
- Dog behaves fearfully during visits to the veterinarian
- Dog enjoys playing with toys
- Dog is friendly to unfamiliar people
- Dog is playful with other dogs
- Dog seeks companionship from people
- Dog behaves submissively (for example, it flips over, avoids eye contact, licks its lips) when greeting other dogs
- Dog adapts easily to new situations and environments
- Dog likes to chase bikes, runners and skateboarders
- Dog is curious
- Dog behaves aggressively in response to perceived threats from people (e.g., being cornered)
- Dog is aloof
- Dog behaves fearfully towards unfamiliar people
- Dog willingly shares toys with other dogs
- Dog is slow to respond to corrections
- Dog behaves aggressively during visits to the veterinarian
- Dog seeks constant activity
- Dog leaves food or items alone when told to do so
- Dog retrieves objects (such as balls, toys, sticks)
- Dog is friendly towards other dogs.
- Dog exhibits fearful behaviors when restrained
- Dog aggressively guards coveted items (e.g., stolen item, treats, food bowl).
- Dog is affectionate

- Dog ignores commands
- Dog behaves aggressively towards cats
- Dog shows aggression when nervous or fearful
- Dog tends to be calm
- Dog behaves fearfully towards other dogs
- Dog is able to focus on a task in a distracting situation (e.g., loud or busy places, around other dogs)
- Dog behaves fearfully when groomed (e.g., nails trimmed, brushed, bathed, ears cleaned)
- Dog is assertive or pushy with other dogs (e.g., if in a home with other dogs, when greeting).
